# Can Cephalopods Vomit? Hypothesis Based on a Review of Circumstantial Evidence and Preliminary Experimental Observations

**DOI:** 10.3389/fphys.2020.00765

**Published:** 2020-07-23

**Authors:** António V. Sykes, Eduardo Almansa, Giovanna Ponte, Gavan M. Cooke, Paul L. R. Andrews

**Affiliations:** ^1^ CCMAR, Centro de Ciências do Mar do Algarve, Universidade do Algarve, Faro, Portugal; ^2^ Department of Aquaculture, Instituto Español de Oceanografía, Centro Oceanográfico de Canarias, Santa Cruz de Tenerife, Spain; ^3^ Department of Biology and Evolution of Marine Organisms, Stazione Zoologica Anton Dohrn, Naples, Italy; ^4^ Department of Life Sciences, Anglia Ruskin University, Cambridge, United Kingdom

**Keywords:** digestive tract, motility, nutrition, *Octopus vulgaris*, regurgitation, *Sepia officinalis*, vomiting, welfare

## Abstract

In representative species of all vertebrate classes, the oral ejection of upper digestive tract contents by vomiting or regurgitation is used to void food contaminated with toxins or containing indigestible material not voidable in the feces. Vomiting or regurgitation has been reported in a number of invertebrate marine species (*Exaiptasia diaphana*, *Cancer productus*, and *Pleurobranchaea californica*), prompting consideration of whether cephalopods have this capability. This “hypothesis and theory” paper reviews four lines of supporting evidence: (1) the mollusk *P. californica* sharing some digestive tract morphological and innervation similarities with *Octopus vulgaris* is able to vomit or regurgitate with the mechanisms well characterized, providing an example of motor program switching; (2) a rationale for vomiting or regurgitation in cephalopods based upon the potential requirement to void indigestible material, which may cause damage and ejection of toxin contaminated food; (3) anecdotal reports (including from the literature) of vomiting- or regurgitation-like behavior in several species of cephalopod (*Sepia officinalis*, *Sepioteuthis sepioidea*, *O. vulgaris*, and *Enteroctopus dofleini*); and (4) anatomical and physiological studies indicating that ejection of gastric/crop contents *via* the buccal cavity is a theoretical possibility by retroperistalsis in the upper digestive tract (esophagus, crop, and stomach). We have not identified any publications refuting our hypothesis, so a balanced review is not possible. Overall, the evidence presented is circumstantial, so experiments adapting current methodology (e.g., research community survey, *in vitro* studies of motility, and analysis of indigestible gut contents and feces) are described to obtain additional evidence to either support or refute our hypothesis. We recognize the possibility that further research may not support the hypothesis; therefore, we consider how cephalopods may protect themselves against ingestion of toxic food by external chemodetection prior to ingestion and digestive gland detoxification post-ingestion. Reviewing the evidence for the hypothesis has identified a number of gaps in knowledge of the anatomy (e.g., the presence of sphincters) and physiology (e.g., the fate of indigestible food residues, pH of digestive secretions, sensory innervation, and digestive gland detoxification mechanisms) of the digestive tract as well as a paucity of recent studies on the role of epithelial chemoreceptors in prey identification and food intake.

## Introduction

In the obligative act of eating, animals expose themselves to the ingestion of food potentially contaminated with toxins, which may not have been detected by vision, olfaction, or gustation prior to swallowing (Davis et al., 1986; Glendinning, 2007); these systems are considered the first line of defense. To avoid or minimize the potential effects of toxins once ingested, they must be detected pre-absorption and/or post-absorption (at low concentrations) and trigger physiological mechanism(s) for rapid removal in bulk from the body either *via* the mouth (vomiting) or anus (diarrhea).

Vomiting in vertebrates describes the forceful ejection of upper digestive tract contents from the body *via* the mouth in a single coordinated action with the animal usually adopting a characteristic posture assumed to optimize the mechanics of ejection and reduces strain on the body musculature (Stern et al., 2011; Andrews and Rudd, 2015). The word regurgitation is sometimes used interchangeably with vomiting, as it can also result eventually in oral expulsion of upper digestive tract contents if combined with “spitting,” but regurgitation should be used to describe movement of previously swallowed solids or liquids only to the buccal cavity. However, regurgitation is used to describe the process by which some mammals and birds (Lecomte et al., 2006) feed their young and by which some birds (e.g., owls) void pellets of indigestible material (Duke et al., 1976).

Vomiting is only one of the strategies by which animals defend against toxins in the food. Other strategies in vertebrates include, for example, vision, taste, smell, learned aversions, ingestion of clay to adsorb toxins, and hepatic detoxification (Davis et al., 1986; Glendinning, 2007; Stern et al., 2011). This hypothesis focuses on the possibility of vomiting or regurgitation in cephalopods, so a detailed discussion of all the potential toxin defensive mechanisms is outside the immediate scope of this paper, and functions of vomiting or regurgitation may extend beyond toxin defense (see below). However, as vomiting and regurgitation are only one of the potential defensive strategies, we consider some of the other mechanisms in the section on Testing the Hypothesis, where the implications should cephalopods eventually be proven to neither vomit nor regurgitate are discussed.

Vomiting or regurgitation has been reported in representative species of all vertebrate classes, although the mechanics differ and the functions extend beyond ejection of toxic food, but a review is outside the scope of this paper, so the reader is referred to the following papers: fish, Andrews and Young (1993); Sims et al. (2000); amphibia, Naitoh et al. (1989); Naitoh and Wassersug (1992), reptiles, Andrews et al. (2000); birds, Duke et al. (1976); and mammals, Horn et al. (2013).

In contrast to the vertebrates, there are relatively few published reports of either vomiting or regurgitation in invertebrates, although the ability of arthropods to regurgitate is well described (e.g., *Apis mellifera*: Chapman, 1969; *Locusta migratoria*: Freeman, 1968; *Schistocerca emarginata*: Sword, 2001). To provide a background, we firstly briefly review the literature on vomiting or regurgitation in marine invertebrates. We then consider why cephalopods may need to vomit, observational evidence, and anatomical and physiological data on the digestive tract to provide insights into potential mechanisms and identify any constraints, which would make either vomiting or regurgitation difficult (e.g., see Horn et al., 2013, for discussion of inability of rodents to vomit). It is important to note that there are no published studies which have directly investigated either vomiting or regurgitation in cephalopods, so we can only include evidence that is supportive of our hypothesis. However, we describe studies using currently available methodologies which could confirm or refute our hypothesis and consider the implications for how cephalopods may defend against toxic foods if our hypothesis is refuted by direct experimental studies. While the focus is on vomiting and regurgitation, collating evidence for our hypothesis has necessitated a detailed examination of a number of aspects of cephalopod digestive tract anatomy and physiology, resulting in identification of a number of knowledge gaps.

## Vomiting and Regurgitation in Marine Invertebrates: Some Examples

We describe examples of vomiting/regurgitation from three classes (*Anthozoa*, *Malacostraca*, and *Gastropoda*) of marine invertebrate solely to illustrate that this ability is not confined to marine vertebrates (see above). Only publications where the authors have themselves referred to an event as either regurgitation or vomiting and have provided a clear description of the phenomenon are reviewed here. In the sections below, we adopt the terminology used in the original publication (i.e., vomiting or regurgitation).

The sea anemone *Exaiptasia diaphana* (cited as *Aiptasia pallida*) regurgitated pellets made from squid meat mixed with an extract of the tunicate *Trididemnum solidum* (Lindquist and Hay, 1995). The tunicate extract contains the secondary metabolites and didemnins, used to chemically defend the larvae from predation by fish.

The mechanics of regurgitation of the foregut contents following feeding have been described in both the red rock crab (*Cancer productus*) and the graceful crab (*Metacarcinus gracilis*, originally referred to as *Cancer gracilis*) in response to exposure to air and reduced salinity (McGaw, 2006, 2007; for review see McGaw and Curtis, 2013). The ejection is by initial relaxation of the foregut with subsequent intense contraction of the foregut muscles (pyloric and cardiac stomach) pushing the material into the esophagus, which is already opened and from where it exits *via* the mouth. In some animals, the entire foregut contents were ejected in a few minutes. As the normal motility cycle in the upper digestive tract is under the control of the stomatogastric nervous system (commissural ganglion, esophageal, and stomatogastric ganglia), it is likely that regurgitation occurs by motor program switching in these ganglia (see below).

The vomiting, regurgitation, and rejection mechanism in the gastropod mollusk *Pleurobranchaea californica* has been studied in detail (McClellan, 1982, 1983; Croll et al., 1984). *Pleurobranchaea*, which had swallowed a mixture of fresh squid homogenate mixed with rotten squid homogenate, expelled it from the body *via* the buccal cavity with the “vomiting phase” lasting 46.1 ± 6.9 s (McClellan, 1982). The author proposed that the initial propulsive force for ejection was provided by contraction of the body wall and gut muscles. Vomiting was accompanied by tonic shortening of the esophagus, but reverse peristalsis of the esophagus was not observed and final ejection from the buccal cavity was due to cyclical movements of the radula. Vomiting was also evoked if fresh squid was mixed with a dilute solution of liquid detergent, a stimulus capable of inducing vomiting in dogs and humans (Weaver and Griffiths, 1969). It was also reported that animals would reject pieces of rubber tube mixed with palatable food, but this ejection differed from vomiting as the ejection was from the buccal cavity and not the stomach. The neural control of swallowing, vomiting, and rejection provides a good example of “motor program switching” as is also the case for vomiting in vertebrates (Stern et al., 2011).

Studies in *Aplysia californica* have shown the egestion of material (seaweed) from the buccal cavity and esophagus (Jing and Weiss, 2001) and reduced feeding behavior when food was paired with a negative reinforcer (Susswein et al., 1986). Egestion of food by modification of the feeding pattern has also been reported in the pulmonate gastropod *Lymnaea* (Elliott and Susswein, 2002).

The above brief survey of published evidence provides clear, but limited, evidence that the ability to void material from the gut *via* the mouth is not confined to vertebrates, but clearly additional studies are required to identify the circumstances under which ejection of previously ingested food or other material occurs in the wild.

## Why is it Important to Know if Cephalopods can Vomit?

The answer to this question is approached by analogy with species in which vomiting or regurgitation is known to be present and by considering components of the cephalopod diet.

### The Diet May Contain Indigestible Components Which Cannot Be Voided in the Feces

Although there are some data on fecal color (Taki, 1941), data on analysis of feces in either captive or wild cephalopods are very rare, so it is not known with certainty what, if any, indigestible material can be voided by this route; but see Bidder (1957, p. 143) and Wells and Wells (1989, p. 220) for rare examples. The presence of small amounts of indigestible material in the feces does not exclude the possibility that the same material is ejected in larger quantities *via* the mouth following digestion of food in the upper digestive tract. Passage of indigestible material beyond the stomach, should it occur, risks obstruction and damage to distal structures with the cecal lamellae being at particular risk together with the typhlosoles in the intestine (particularly prominent in *Nautilus*; Budelmann et al., 1997).

So what evidence is there for the presence of indigestible material in the cephalopod digestive tract? In the earliest published description of the digestive tract of *Nautilus*, Owen (1832, p. 24) commented “*The whole alimentary canal was filled with fragments of Crustaceans among which portions of branchiae, claws, and palpi were distinctly recognisable…[…] The crop in particular was densely filled with these fragments*.” Examination of the stomach contents in wild caught *Sepia officinalis* (Guerra et al., 1988) also revealed the presence of fragments of crustacean exoskeleton, bones, and scales from fish and beaks from sepioid cephalopods. Pieces of crustacean exoskeleton, with attached soft tissue cleaned, were present in 90% of the cuttlefish examined and in animals >100 mm dorsal mantle length (DML) pieces of skeletal material ~5–17 mm long and ~2.5–5 mm wide were found; the esophagus diameter was 2–3 mm in animals in this size range. A further example is provided by the deep-sea octopus *Graneledone c.f., boreopacifica*, in which the gut was found to contain gastropod shells, shell fragments, polychaete bristles, and jaws (Voight, 2000). Polychaetes (*Hermione hystrix*) with indigestible cetae are part of the diet in octopods (including *Octopus vulgaris*; Nixon and Budelmann, 1984).

In *O. vulgaris* fed on crabs or dead fish, gill leaflets were found in the crop 2 h post prandial (Altman and Nixon, 1970), and in *O. vulgaris* fed on 10 g of sardine, fish bones were visible in the stomach 3 h after feeding when the stomach contents had a clay-like appearance (Andrews and Tansey, 1983). Some sardine bones were also observed in the intestine of animals killed >1 h post feeding. The cephalopod stomach does not secrete hydrochloric acid, which contrasts with the stomach in most vertebrates in which the gastric pH is between 1 and 2 (Babkin, 1950). It is the acid which is responsible for digestion of bone in the vertebrate diet. The pH of stomach contents of *Nautilus pompilius* and *O. vulgaris* is only mildly acidic with a pH of ~5.1–5.8 (Mangold and Bidder, 1989, p. 351, Table XVI), consistent with the acidic pH optima of many digestive enzymes in cephalopods (Linares et al., 2015; Gallardo et al., 2017).

Overall, while there is a body of evidence showing that the crop and stomach of cephalopods contains indigestible residues after the soft tissue has been removed, the fate of this material remains unknown. Quantifying the ability of cephalopod digestive tract secretions with a pH 5–6 to degrade small fish bones, scales, and fragments of crustacean exoskeletons at body temperature will provide part of the answer to their fate in the digestive tract. However, detailed studies of the composition of feces (as proposed by Ponte et al., 2017) are also required to enable a more informed conclusion to be reached about voiding by defecation vs. the possibility that indigestible residues are voided by vomiting or regurgitation.

### Cephalopods May Ingest Food Contaminated With Toxins Known to Induce Vomiting in Vertebrates

The most likely source of an emetic agent (i.e., a vomit-inducing chemical) is the food cephalopods eat (mollusks, crustacea and fish; see Villanueva et al., 2017, for review), which may contain harmful algal blooms producing a range of toxins as secondary metabolites. It is estimated that at least 100 species of microalgae produce structurally diverse toxins and many of these cause nausea and vomiting in humans (Sobel and Painter, 2005; Berdalet et al., 2015).

Of particular relevance to cephalopods is domoic acid, shown to accumulate in multiple tissues in cephalopods (Lopes et al., 2013, 2018), and which can induce vomiting in humans (see Pulido, 2008, for review) and Cynomolgus monkeys (Tryphonas et al., 1990). Although there is no evidence that consumption of mussels contaminated with domoic acid alters food intake in *O. vulgaris* (Lopes et al., 2013), there are also no studies examining a wide dose-range of domoic acid on the functioning of the digestive tract either *in vivo* or *in vitro*. Electrophysiological studies of the effect of domoic acid on neurotransmission in slices of *O. vulgaris* vertical lobe demonstrated potent effects on the AMPA-kainate type glutamate receptor with a calculated EC_50_ of 0.28 ± 0.05 μM, making it the most potent of the agonists used (domoic acid > SYM2208 > > CNQX >> ʟ-glutamate > > kynurenic acid; Langella, 2005). Domoic acid exposure resulted in irreversible neurotoxicity in the vertical lobe slices (Langella, 2005). There is evidence for glutamate receptors (both AMPA-kainate and NMDA type) in cephalopod central and peripheral neural tissue (for references see Lima et al., 2003; Di Cosmo et al., 2006; Lee et al., 2013). Transcriptomic evidence supports the presence of glutamate receptors in the gastric ganglion of octopus (Zarrella, et al., 2019; Ponte, personal communication) but they have not been demonstrated in the enteric nervous system although this seems very likely, making the digestive tract neural control mechanisms a potential target for toxic effects of domoic acid. As domoic acid is widely distributed in visceral tissues (e.g., digestive gland, posterior salivary glands, kidney, gills, and systemic heart; Costa et al., 2005) and the brain (Lopes et al., 2018), it is highly likely that the neurones in the wall of the gut and the gastric and buccal ganglia will also be exposed. The gastric ganglion has a variety of putative neurotransmitters and receptors (Andrews and Tansey, 1983; Baldascino et al., 2017), so even if glutamate receptors are present and the associated neurones are damaged, a total loss of functionality is unlikely but the ability of the gastric ganglion to coordinate digestive tract motility may be disrupted.

Saxitoxin accumulates in cephalopod tissues (Lopes et al., 2014), acts on voltage-gated sodium channels (Wiese et al., 2010), and is emetic in humans (James et al., 2010), but there is no evidence that it has deleterious effects on cephalopods (Lopes et al., 2014).

Although the above section has focused on algal toxins there are at least two other potential sources of toxins which could act as an emetic stimulus.

- *Plastics*. Ingestion of plastics has been demonstrated in a diverse range of marine species (e.g., Law, 2017), particularly predators (e.g., Nelms et al., 2018) including one species of cephalopod, the jumbo squid *Dosidicus gigas* (Braid, et al., 2012; Rosas-Luis, 2016). Examination of gastric contents in *D. gigas* revealed the presence of fishing line, plastic pellets, and pieces of polyvinyl chloride (PVC; ~1 cm) fishing floats as well as rocks, sand, and plant matter (Braid, et al., 2012; Rosas-Luis, 2016). Larger pieces of plastics could evoke vomiting or regurgitation by triggering mechanisms detecting indigestible food residues (see below) and smaller pieces could accrete, leading to digestive tract obstruction (e.g., bezoars in humans and cats). A further possibility is that the chemicals released from the plastics could themselves act as emetic stimuli. For example, phthalates used as plasticizers in PVC production can cause nausea and digestive tract discomfort in humans (Diaz, 2015).
- *Heavy metals*. Many heavy metals in trace quantities are essential for normal operation of a number of metabolic processes (e.g., enzymes), but heavy metals can also be toxic to the organism. The digestive gland in cephalopods accumulates a range of heavy metals and is implicated in their detoxification (e.g., Penicaud et al., 2017; Rodrigo and Costa, 2017). However, a number of heavy metals cause acute nausea and vomiting in humans when ingested in higher concentrations including mercury, copper, and zinc (Cushny, 1918). Copper sulfate has been extensively as an emetic in experimental studies of emesis in vertebrates to induce emesis by gastric administration including in fish (see Table 1 in Tiersch and Griffiths, 1988).

### Vomiting Can Be a Symptom of Disease

Vomiting, particularly if chronic, is recognized as a symptom of disease in both human and veterinary medicine. In the context of animals in laboratory-based research (e.g., under Directive 2010/63/EU; Fiorito et al., 2015), vomiting or regurgitation would be an important indicator of poor welfare or an adverse reaction to a procedure, particularly one involving pharmacologically active agents as occurs frequently in mammals (e.g., Percie du Sert et al., 2012). Regurgitation was included in the list of possible indicators of ill health and poor welfare in cephalopods (see Table 5 in Fiorito et al., 2015). The possibility that cephalopods experience nausea (or a functionally equivalent negative hedonic sensation; Stern et al., 2011) should not be excluded particularly in view of the recent discussions of the capacity of cephalopods to experience pain (e.g., Sneddon et al., 2014; Sneddon, 2015; Key and Brown, 2018).

In attempting to answer the question “*Why it is important to know if cephalopods can vomit?*” we have drawn on knowledge of the functions of vomiting and regurgitation in other species and considered how they could apply to cephalopods. Assuming that cephalopods are able to vomit or regurgitate, the most likely functions are periodic ejection of indigestible material (e.g., crustacean exoskeleton pieces, fish bones and scales, and plastic fragments) and acute ejection of toxic food before the toxin can be absorbed in sufficient quantity to have systemic toxic effects. Obviously, these functions will depend on the diet of the species, so it is conceivable that not all species may have the capacity to either vomit or regurgitate, so formal investigation may require studies in representatives of at least each sub-class.

## Behavioral Observations Supporting the Presence of Vomiting in Cephalopods

The data summarized below are primarily anecdotal reports from the literature but with limited support from incidental observations by the laboratory of two of the present authors. All the reports below are from cephalopods in captivity. In the wild, vomiting/regurgitation may be very difficult to observe (see the section on Testing the Hypothesis-iv below), but we are unaware of any published reports claiming to have observed this phenomenon in the wild and we have not identified any studies systematically investigating the ability, or not, of cephalopods to either vomit or regurgitate in the laboratory.

Immersion of the Caribbean reef squid, *Sepioteuthis sepioidea*, in the anesthetic agent magnesium sulfate (3 and 4% in filtered sea water) evoked “regurgitation of stomach contents” in some animals (García-Franco 1992, p. 121) and most animals defecated. The same reaction was not observed when the animals were immersed in a solution of magnesium chloride in sea water (2 and 3%). Vomiting or regurgitation and defecation in response to exposure to putative general anesthetic agents has been observed in juvenile/adult *S. officinalis* if they were not fasted for 24 h prior to exposure to the anesthetic agent (Sykes, unpublished observations).

There is an anecdotal report of the occurrence of vomiting/regurgitation in *Enteroctopus dofleini* during net-feeding in captivity (Gleadall, personal communication cited in Andrews et al., 2013).

Vomiting- or regurgitation-like events have been observed in free swimming juvenile/adult *S. officinalis* during which the animals appear to adopt a characteristic posture (Sykes, unpublished observations). Although data on the incidence have not been gathered, the impression of those who inspect the animals daily is that it is not a rare event. The accompanying video clips (taken using Sony Action Cam HDR-AS10) from intermittently monitored tanks of breeding cuttlefish fortuitously captured the behavior accompanying a “spontaneous” suspected vomiting-like event in cuttlefish^1^. Similar events were observed directly by technical staff in fed animals.

Over a 2-year period, four adult (weight 1–2.5 kg) *O. vulgaris* have been observed to have a vomiting- or regurgitation-like event following feeding a crab, but the time after feeding is not known as the suspected vomit was found when the tank was inspected the next day and in one case the animal ejected material when it was moved between tanks (Almansa, unpublished observation). The latter suggests that vomiting could be induced by handling in fed octopus, in a similar way to *S. officinalis* (see above). Suspected vomit had the appearance (mucoid, viscid, and particulate) and color (brown) of partially digested gastric contents and could be readily distinguished from feces, which were in ropes and were a lighter orange, white, or brown color (according to the diet) compared to gastric contents.

The above observations reporting vomiting- or regurgitation-like events in cephalopods from the literature and unpublished observations should all be treated with caution until confirmed by more systematic studies designed specifically to investigate this phenomenon (see the section on Testing the Hypothesis).

Additional indirect support for our hypothesis comes from a paper, published while this paper was initially under review, analyzing fossilized regurgitalites (orally ejected stomach contents) containing aptychi (calcitic lower jaws of ammonites) from late Jurassic Solnhofen deposits (Hoffmann et al., 2019). From a detailed study of the regurgitalites, data on fossilized cephalopod digestive tract contents and other fossils found in the same or related deposits, the authors built a case (using some of the same literature reported here) that coleoid cephalopods and most likely vampyropods were the predators responsible for the origin of the regurgitated ammonite remains.

## The Possible Mechanics of Vomiting or Regurgitation in Cephalopods

Here, we consider if it is theoretically possible for cephalopods to vomit or regurgitate by focusing on *S. officinalis* and *O. vulgaris* as relevant anatomical and physiological data are available in the literature, which we supplement by some additional data.

The key issues are: (a) Where could the force required to expel the contents from the crop/stomach into the esophagus originate? (b) What are the resistances to the movement of material?

### Anatomical Considerations

#### Buccal Mass

The buccal mass is the final structure through which material if ejected from the crop/stomach *via* the esophagus would need to transit, prior to ejection from the body. The buccal mass is formed from the chitinous beak, the mandibular muscles (superior, inferior, and lateral; Boyle et al., 1979), the lateral buccal palps, and the radula and associated bolster muscles (see Messsenger and Young, 1999, p. 163, Figure 1). To minimize resistance to material emerging from the esophagus, into the pharynx and subsequently the mouth, the lateral palps would need to be retracted and the beak opened as occurs at the start of the “bite cycle” (described in *O. vulgaris* by Boyle et al., 1979, p. 59, Figure 6). *In vitro* studies of the buccal mass in *O. vulgaris* showed that the opening phase of the bite cycle lasted 5.6 ± 2.3 s during a spontaneous bite; this would provide sufficient time for material to be ejected if opening was coordinated with the arrival of crop/stomach contents into the pharynx. Cyclical activity of the radula plays a role in egestion in *P. californica*, but we are unable to comment whether the radula working in the opposite way to the “backward-forward,” chain saw-like, motion (Messenger and Young, 1999) utilized in swallowing food could be involved in the ultimate ejection of material. The radula is not critical in swallowing (Boyle et al., 1979), so even if it is involved in ejection its role may not be essential.

The movements of the buccal mass are regulated by a “programme of actions” (Boyle et al., 1979) generated in the inferior buccal ganglion, which provides the innervation to the muscles (Young, 1971). The inferior buccal ganglion also innervates the esophagus and crop, and the gastric ganglion is also innervated by the visceral nerves (Young, 1967). Thus, all the key structures, which may be involved in vomiting or regurgitation (buccal mass, esophagus, crop, and stomach) are innervated either directly or indirectly (*via* the gastric ganglion) from the inferior buccal ganglion. This makes the buccal ganglion a likely site for the genesis of a vomiting or regurgitation “motor program” initiated in response to inputs from the superior buccal lobe of the brain, which itself may be innervated from the subvertical lobe (Young, 1971). Potential pathways are reviewed in more detail in the “Discussion” section.

#### The Length and Diameter of the Esophagus

To reach the buccal cavity, gastric or crop contents must transit the full length of the esophagus. Measurements taken immediately *post mortem* of the length of the esophagus *in situ* in fresh specimens show that the length is 68.9 ± 8.5% of the DML in *S. officinalis* (mean ± SD: body weight 367.5 ± 82 g; DML 140 ± 7 mm, *N* = 10) and 35.5 ± 2.1% in *O. vulgaris* (mean ± SD: body weight 890 ± 57 g; 130.7 ± 4.3 mm, *N* = 7). Based on a dissection photograph (see Guerra, 2019, p. 24, Figure 3.11) of *Loligo vulgaris*, we estimate that the esophagus to stomach distance is at least 50% of the DML and the same appears true for *Loligo pealei* (see Berk et al., 2009, p. 2, Figure 1f). The relatively narrow diameter of the esophagus at the junction of the crop/stomach is a resistance to retrograde flow of contents. If the esophagus in either octopus or cuttlefish acts as a purely passive conduit for the passage of material ejected by crop/gastric contractions, then digesta will not be visible in the beak until the volume ejected from the crop/stomach exceeds the volume of the esophagus; for *O. vulgaris* and *S. officinalis* (in the above weight range and using the mean length), this is calculated to be 0.5 and 1.2 ml, respectively.

#### Sphincters

**Figure 1:**
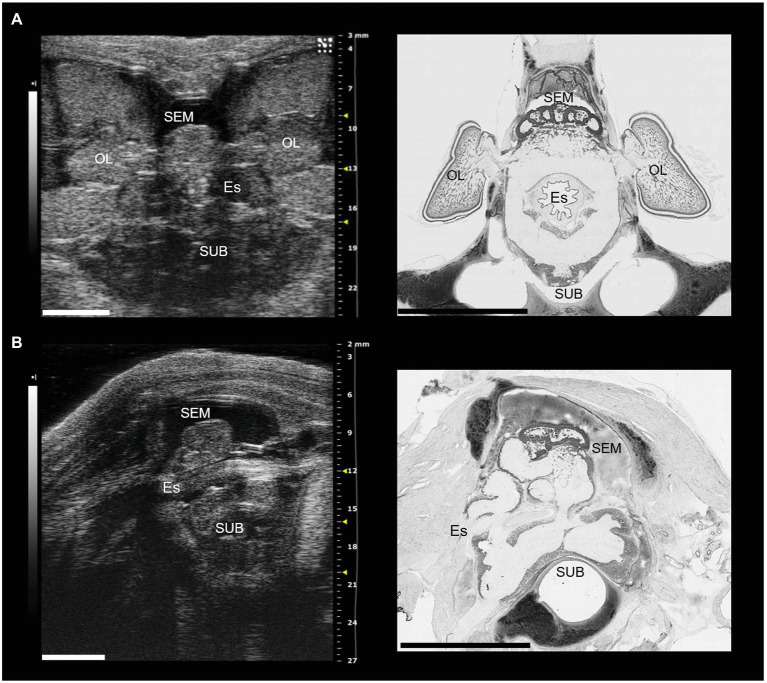
The brain of *Octopus vulgaris* as it appears using sonographic scanning (for additional details see Grimaldi et al., 2007) and histological sections after Nissl staining (in black and white). **(A)** Ultrasound examination (left) of the entire cerebral mass in the coronal plane, and the corresponding histological section (right). The optic lobes are visible bilaterally and appear connected through the optic tracts to the central brain (supraesophageal mass, SEM). The subesophageal mass (SUB) lies ventrolateral to the esophagus (Es). **(B)** Ultrasound examination of the central brain in the sagittal plane (left), and the equivalent histological section (right). The two masses (SEM and SUB: dorsally and ventrally, respectively) are clearly visible with the esophagus in the middle. OL, optic lobe; Es, esophagus; SEM, supraesophageal mass; SUB, subesophageal mass. Scale bars: 5 mm.

There is no evidence in the literature for the existence of a sphincter (i.e., a structure formed from a thickened layer of circular muscle, resulting in a region with elevated pressure compared to adjacent regions) at the junction between the pharynx and the esophagus (e.g., Boyle et al., 1979; Guerra et al., 1988; Messenger and Young, 1999). However, in the coleoid cephalopods the esophagus passes between the supra-esophageal and sub-esophageal masses of the brain (Figure 1) located dorsal and ventrally, respectively (see e.g., Grimaldi et al., 2007). This anatomical organization will limit the size of pieces of food, which can pass to (swallowing/ingestion) or from (regurgitation/vomiting) the crop/stomach. In *Nautilus*, the brain is less well-developed and the circumesphageal connections are less likely to constrain bolus size.

At the junction between the esophagus and its entry into the crop in *O. vulgaris*, the circular muscle has a sphincter-like appearance, fluid in the crop did not reflux readily into the esophagus *post mortem* and resistance to the passage of a cannula was reported (Andrews and Tansey, 1983). Best and Wells (1983) also reported that fluid in the crop of *O. vulgaris* did not pass readily into the esophagus. However, there is no physiological evidence demonstrating a zone of elevated pressure between the esophagus and crop in octopus or the esophagus and stomach in cuttlefish. Still, Boucaud-Camou and Boucher-Rodoni (1983, p. 164) comment that “*sphincters enable both the caecum and the stomach to be isolated from the rest of the digestive tract*.” This statement was not referenced, but we have concluded that it refers to observations from *S. officinalis* published in Boucaud-Camou (1977) and/or from drawings in the anatomical monograph of *S. officinalis* by Tompsett (1939, Figure 46) showing sphincters at the esophagus-stomach, stomach-caecum, and stomach-intestine junctions. A recent guide to the functional anatomy of cephalopods does not describe sphincters between any of these structures in the section on the digestive tract (Guerra, 2019), and this is also the case for a chapter including a survey of the histology of the digestive tract (Anadon, 2019), and a research paper describing the anatomical and histochemical features of the *O. vulgaris* digestive tract (Fernandez-Gago et al., 2019). More detailed histological and functional studies are required to resolve the issue of the presence of sphincters between key regions of the cephalopod digestive tract.

Direct observations of the *O. vulgaris* digestive tract *in vitro* and *in vivo* have identified a reciprocal exchange of contents between the crop and stomach with functional evidence indicating that in octopuses (both adults and paralarvae), the crop (when present) and the stomach operate as a single functional unit (Andrews and Tansey, 1983; Nande et al., 2017). In the absence of a sphincter, the flow of contents between these regions of the digestive tract will depend upon the intra-luminal pressure differential between adjacent regions, the diameter of the lumen at the junction between the adjacent regions, the direction (aboral or oral) and amplitude of the peristaltic contractions, and the viscosity of contents (see below).

### Physiological Considerations

#### Motility in the Esophagus

Peristaltic activity in the esophagus moving contents in an aboral direction has been reported in *O. vulgaris* (Andrews and Tansey, 1983) and *Doryteuthis pealeii* (Wood, 1969) *in vitro*. Although retrograde peristalsis has not been reported in cephalopods, there is no *a priori* reason why it could not occur as is the case in the mammalian small intestine immediately prior to vomiting (Lang, 2016) and the esophagus of birds during regurgitation (Duke et al., 1976). Retro-peristalsis would provide a mechanism for ejection of material delivered from the crop or stomach into the esophagus. For this to occur the enteric nervous system pathways for the oro-anal peristalsis need to be overridden and this is most likely to occur by extrinsic nerves from the buccal or gastric ganglia. Longitudinal shortening of the esophagus by contraction of the inner layer of longitudinal obliquely striated muscle (Budelmann et al., 1997) would also facilitate expulsion of material from the crop/stomach.

In *Nautilus*, Owen (1832) reported that the esophagus was only three-fourth of an inch (~2 cm) long before entering the relatively large crop (known to contain indigestible food residues), but the size of the animal was not reported. In diagrams of the *Nautilus* digestive tract [e.g., Figure 1 in Westermann et al. (2002, p. 1618)], the esophagus is relatively short when comparing its length in similar diagrams of the digestive tract in other cephalopods and could shorten further by longitudinal contraction facilitating ejection of crop contents if retropulsive contractions occurred in the crop.

#### Motility in the Crop and Stomach

Because of the differences in digestive tract morphology between cuttlefish, squid, and octopus digestive tracts, we will discuss them separately. In cuttlefish^2^ and squid tonic contraction of the stomach could propel gastric contents directly into the esophagus from where retro-peristaltic contractions could transport them to the buccal cavity. Orally directed peristaltic contractions (8–13 mm/s) have been reported in the stomach of the squid *Doryteuthis pealii* (Wood, 1969) and such contractions could push material toward the esophagus, but a regular cycle of oral/aboral contractions would be expected as a component of normal gastric motility to triturate the food and mix it with digestive secretions.

**Figure 2:**
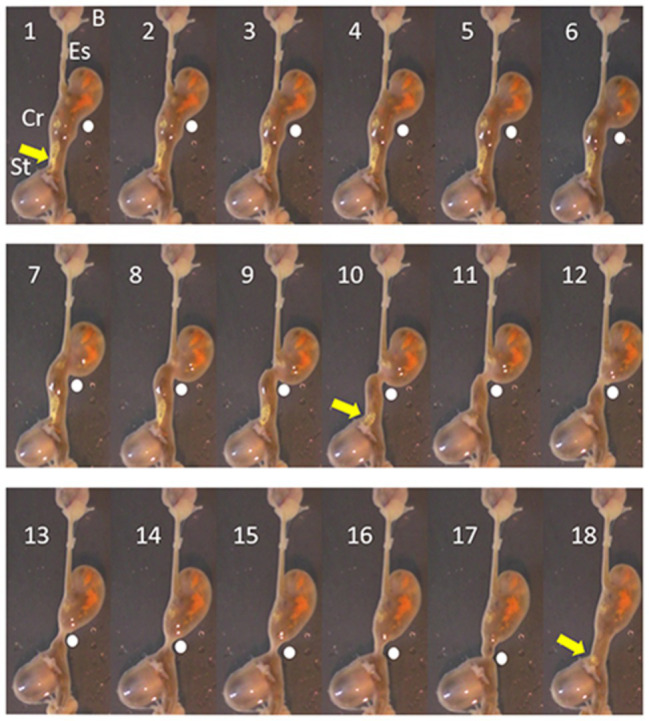
Pictures taken each second (1–18 s) from a video recording of the digestive tract of *O. vulgaris*, *in vitro* (in sea water gassed with air). The animal had been fed a crab 1 h before killing (see Fiorito et al., 2015). The sequence shows the progression of a peristaltic wave (indicated by a white dot) from close to its origin and progressing aborally to the crop/stomach junction. The pressure created by the contraction moves material (yellow arrow) from the crop to the stomach and it is no longer visible after Frame 10. In Frame 16, the crop contraction reaches the stomach and in frames 17 and 18, the contraction subsides allowing material (yellow arrow) to reflux from the stomach toward the crop. The vertical axis of the frames is ~8 cm. B, beak; Es, esophagus; Cr, crop; and St, stomach.

**Figure 3:**
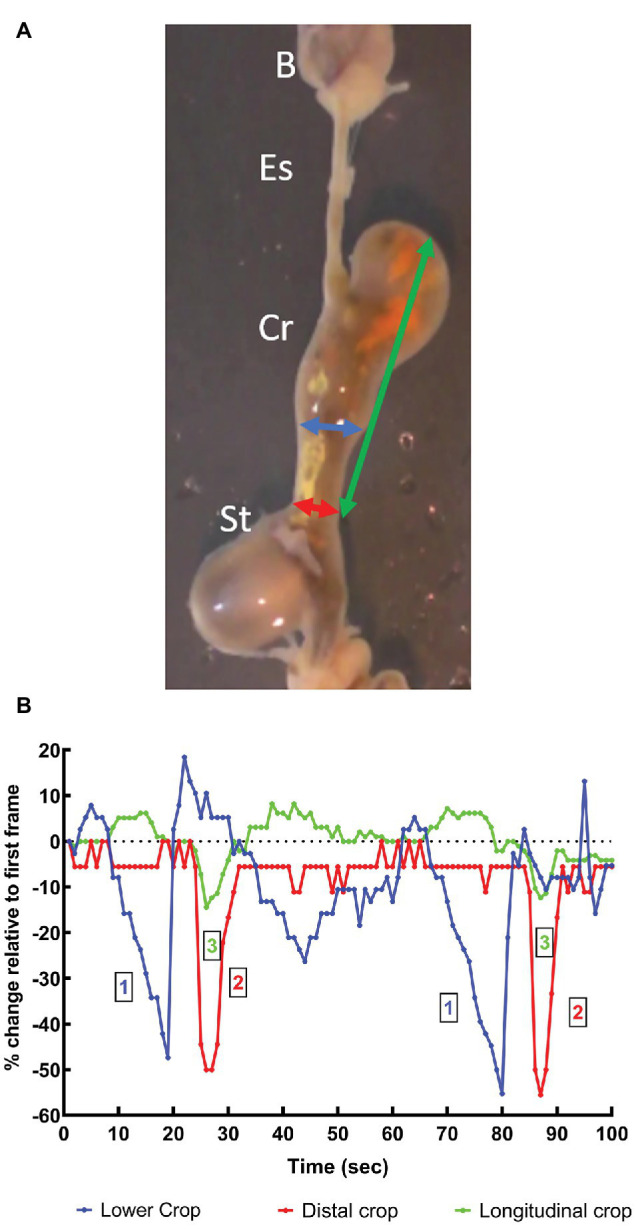
**(A)** Video frame showing the beak, esophagus, crop, and stomach *in vitro* (see above text for details) from an *O. vulgaris* fed a crab 1 h before killing. The vertical axis of the frame is ~8 cm. Arrows indicate the measurements made of the apparent diameter of the lower crop (blue), distal crop (red) at the crop-stomach junction and the length of the crop (green). B, beak; Es, esophagus; Cr, crop; St, stomach. **(B)** Measurements of the changes in the dimensions of the crop made *in vitro* from an animal fed a crab 1 h before killing. The measurements defined above are made each second from 100 s of video recording (see Figure 2) and show two cycles of contraction. All measurements are expressed as a percentage change relative to those taken at *t* = 1 s. The two contractile cycles closely duplicate each other ~1 min apart. The graph shows the wave of contraction of the lower-crop (1) passes to the distal crop (2). The contraction of the distal crop is accompanied by longitudinal shortening of the crop (3). Note that both the lower and distal crop change apparent diameter by ~50% during the passage of the peristaltic contraction.

In octopus, tonic contraction of the longitudinal and circular muscle of the crop would push contents into the esophagus, if this was accompanied by contraction of the stomach and relaxation of the relevant sphincters (if present). Contraction of the stomach can propel semi-solid material into the crop but the impact of stomach contractions on crop pressure will be low because of the relatively thin walls of the crop and its ability to relax to accommodate contents. *In vitro* the digestive tract of *O. vulgaris* shows peristaltic contractions originating in the crop caudal to the entry of the esophagus, sweeping along the length of the distal crop and pushing *boli* of food into the stomach with the crop/stomach junction narrowed as the bolus enters the stomach (Figure 2). The passage of the peristaltic contraction over the distal crop is accompanied by longitudinal shortening of the crop (Figure 3). However, once the constriction of the circular muscle between the crop and stomach relaxes gastric contents reflux into the crop accompanied by a gastric contraction (Figure 2). This cycle is repeated as the next crop contraction passes to the stomach (Figure 3). This observation shows that there is no barrier to the passage of gastric contents into the crop other than the relative contractile activity of the adjacent regions. The contractions of the stomach combined with transient inactivity in the crop are clearly capable of pushing semi-digested food into the crop, which would be a preparatory phase for ejection *via* the esophagus. *Note*: this study used tissue removed *post mortem* from animals killed according to current guidelines (Fiorito et al., 2015) and is not covered by EU/2010/63 (Baldascino et al., 2017). The digestive tract was placed in sea water at ambient temperature (~22°C) and gassed with air; tissue remained active for at least 1 h.

We hypothesize that material could be moved into the esophagus in octopus by: (i) tonic contraction of the stomach both pushing gastric contents into the crop and preventing their return; (ii) cessation of orthograde peristalsis in the crop and its replacement by retrograde contractions originating at the crop/stomach junction, and (iii) gastric/crop contents are pushed into the relaxed posterior esophagus from where retrograde contractions of the circular muscle of the esophagus propel the *boli* to the buccal cavity for ejection possibly involving the radula.

#### Pressure Generation in the Mantle

Forceful, rapid contraction of the mantle muscle could cause an increase in pressure within the crop/stomach to move contents into the esophagus (and possibly beyond) if the exits from the mantle (opercula and funnel) were transiently occluded; a situation very similar to the events occurring during jetting locomotion when the intra-mantle pressure rises rapidly and is then released by opening the funnel. Trueman and Packard (1968) recorded intra-mantle pressure pulses of duration 150 and 180 ms in *S. officinalis* (250 g body weight) and *L. vulgaris* (350 g), respectively, and of 200 ms in *Eledone moschata* (600 g) and 600 ms in *O. vulgaris* (370 g). The peak pressures recorded ranged from ~100 cm H_2_O (73.5 mmHg) in *S. officinalis* to ~400 cm H_2_O (294 mmHg) in *E. moschata*. These pressures are in the range of the mean and peak pressures recorded in the human stomach during vomiting (Iqbal et al., 2008), when the main expulsive force is provided by contraction of the diaphragm (excluding crura) and the anterior abdominal muscles compressing the stomach (Andrews and Rudd, 2015).

Overall, we consider it unlikely that mantle contraction would be responsible for ejection of contents as the pressure pulse is very brief and the location of the crop and stomach makes efficient compression unlikely.

#### The Physical Nature of the Crop/Stomach Contents

The force required to move crop or gastric contents will depend upon their physical nature. Although there have been detailed analyses of upper digestive tract contents in cephalopods, their focus has been on identifying prey types and indigestible residues (see above) rather than on establishing content characteristics such as viscosity and size and rigidity of solid matter, which would provide insights into the forces needed for ejection. Measurement of viscosity of crop/stomach contents at various stages of digestion would be useful for theoretical modeling of the forces required for vomiting/regurgitation in cephalopods. However, such modeling is likely to be challenging as full mathematical modeling of defecation in penguins (*Pygoscelis antarcticus* and *Pygoscelis adeliae*) was confounded by the inability to measure the viscosity of fecal samples due to the presence of crustacean cuticle, fish bones and scales, and other solid fragments (Meyer-Rochow and Gal, 2003).

## Discussion

The above review of the literature supplemented by limited observations of behavior *in vivo* and the digestive tract *in vitro* lead us to hypothesize that cephalopods are likely to have the ability to vomit or regurgitate. Below, we summarize the proposed mechanics and discuss the physiological mechanisms to show that the key components of a plausible mechanism are present before proposing how the hypothesis can be tested and the implications if the hypothesis is unsubstantiated by further studies.

### A Conceptual Model of Vomiting or Regurgitation in Cephalopods

#### Mechanics of Ejection

We propose that the most likely way in which contents of the stomach or crop could be ejected is the following: (i) Contraction of the stomach pushes contents into the crop (when present), which is expected to have aboral peristalsis inhibited and tone reduced to accommodate material; (ii) in species lacking a crop, contraction of the stomach will push material directly into the esophagus, providing that the esophagus/stomach junction or sphincter is relaxed and sphincters (if present) between the stomach and intestine/cecum are constricted. The stomach in cuttlefish (as represented by *S. officinalis*) and squid (as represented by *L. vulgaris*) are more sacculated and have thinner muscle compared to the “gizzard-like” stomach in octopus, making it possible that these types of stomach have retrograde peristalsis as reported for *D. pealii* by Wood (1969); (iii) in species with a crop, it is proposed that retrograde contraction of the crop pushes material into the esophagus. While relatively little is known of the control of peristalsis in the cephalopod digestive tract, it is likely that it is coordinated by the myenteric plexus neurons of the enteric nervous system described by Alexandrowicz (1928) as is the case in vertebrates (Furness, 2006). The hypothesized retrograde contraction leading to retropulsion of contents would need to overcome any resistance at the esophagus/crop junction (or the esophagus/stomach junction in squid and cuttlefish) unless the muscle in the junctional zone is relaxed; and (iv) as crop/stomach contents enter the esophagus retrograde peristaltic contractions would propel it to the buccal cavity for ejection possibly involving the radula (c.f., opisthobranch mollusks discussed above) or buccal musculature with the beak open (see section Buccal Mass above).

#### Coordination of the Mechanical Events

The gastric ganglion in cephalopods is located at the junction of the crop/esophagus, stomach, cecum, and intestine, so it is ideally located to coordinate motility between the various regions *via* nerves projecting to each region (Young, 1967; see also Figure 1 in Baldascino et al., 2017). In *O. vulgaris*, the gastric ganglion has been shown to be involved in the control of the crop and stomach movements (Andrews and Tansey, 1983). The diversity of putative peptide and non-peptide transmitters and receptors in the gastric ganglion, its relatively large size, and complex internal organization support the proposal that it has an important function in coordinating the functions of the various regions of the digestive tract (Andrews and Tansey, 1983; Baldascino et al., 2017). It is proposed that the motility of the stomach, crop, and esophagus required for ejection of material will be coordinated by the gastric and inferior buccal ganglia, in the same way that the stomatogastric system coordinates comparable events in *Pleurobranchea* and *Aplysia* (see above).

The gastric ganglion is implicated in the control of digestive tract motility post-prandially in cephalopods but how this occurs has not been investigated (Andrews and Tansey, 1983; Baldascino et al., 2017). By analogy with the stomatogastric nervous system in crustacea and opisthobranch mollusks (see above), control involves microcircuits, but changes in activity of such microcircuits can lead to a switch from ingestive to egestive behavior (Jing et al., 2007; Daur et al., 2016). The neural activity of the stomatogastric ganglion in crab is subject to modulation by a range of amines and peptides delivered in the circulation (Marder, 2012). In *Aplysia*, a command-like interneuron, which usually elicits food ingestion has its activity changed to evoking egestive behavior by neuropeptide Y (Jing et al., 2007). The presence of neural circuits for both ingestion and egestion in crustacea and opisthobranch mollusks indicates that both occur in the wild as well as in response to experimental stimuli. Modulation of microcircuits in the cephalopod gastric or the inferior buccal ganglion by a neuroactive agent (including toxins) arriving in the blood and leading to motor program switching provides a plausible mechanism by which vomiting or regurgitation could be initiated.

#### Triggering the Process

If one of the functions of the hypothesized vomiting or regurgitation is ejection of indigestible material, then this is most likely to be detected by mechanoreceptors in the muscle of the crop or stomach, activated by stretch as the material accumulates. Mechanoreceptive afferents sensitive to stretch and contraction of the mammalian stomach are well characterized (e.g., Iggo, 1957; Andrews et al., 1980; Page et al., 2002). There is no neurophysiological evidence for mechanoreceptive afferents in the cephalopod digestive tract but indirect evidence is provided by: (i) a relationship between the tendency to attack and crop distension in *O. vulgaris* (Young, 1960) and (ii) limited histological evidence for the presence of sensory cells in the upper digestive tract of octopus (Botar, 1967). In gastropod mollusks, distension sensitive receptors have been implicated in regulation of food intake with section of the stomatogastric nerves innervating the esophagus and crop, leading to hyperphagia in *Pleurobranchaea* (Croll et al., 1987). Neurophysiological studies are required in cephalopods to investigate the presence of afferent nerves in the digestive tract.

Studies in mammals have demonstrated the presence of visceral afferents in the vagus nerve with receptive fields in the digestive tract mucosa sensitive to mechanical stimulation of the mucosa by stroking (Page et al., 2002); such receptors, if present in cephalopods, would be suited to detection of abrasive indigestible material (e.g., crustacean skeletons and plastic fragments); however, they would not function in areas where there is a thick chitinous covering of the epithelium (e.g., thick stomach muscle in *O. vulgaris*). Mucosal receptors in the mammalian stomach and intestine also act as chemoreceptors detecting nutrients (e.g., glucose, lipids, and proteins; Brookes et al., 2013; Williams et al., 2016) and irritants including substances capable of induced nausea and vomiting when given luminally (e.g., copper sulfate: Endo et al., 1995; TRPA1 agonists: Bellono et al., 2017; and hypertonic NaCl: Clarke and Davidson, 1978). The anatomical correlate of the mucosal receptor is an enteroendocrine cell releasing neuroactive agent (e.g., 5-hydroxytryptamine, substance P, and cholecystokinin) at its basal surface to activate receptors located on vagal afferents terminating in close proximity (Grundy, 1988; Reybold, 2010; Powley et al., 2011; Bohorquez et al., 2015; Bellono et al., 2017); this is termed “neurocrine signaling.”

The existence of enteroendocrine cells in the cephalopod digestive tract with afferents terminating in proximity has not been investigated, but neurites arising from bipolar cells in subepithelial plexus supplying the mucosa are argued to be sensory (Alexandrowicz, 1928).

The triggering of vomiting or regurgitation by activation of digestive tract afferents (projecting either to the peripheral ganglia or to the brain) is the most likely mechanism but other options include: (i) a direct effect of a systemic toxin (e.g., domoic acid) on the gastric ganglion, inferior buccal ganglion or possibly the brain [e.g., superior buccal lobe; c.f., centrally acting emetics in mammals; Stern et al. (2011)] and (ii) release of a hormone from the digestive tract mucosa in response to an ingested toxin or irritant resulting in an effect on the inferior buccal ganglion, gastric ganglion, or brain (e.g., superior buccal lobe).

## Testing the Hypothesis

Four pieces of evidence reviewed above support our hypothesis that cephalopods are likely to have the capacity to vomit/regurgitate: (1) the mollusk *P. californica* is able to vomit/regurgitate with the muscular and neural mechanisms well characterized. We acknowledge that gastropods and cephalopods are phylogenetically distant classes but there are similarities in digestive tract morphology and innervation, which support the mechanisms we propose for vomiting or regurgitation in cephalopods; (2) a rationale for the requirement to vomit or regurgitate is presented based upon voiding indigestible food residues, which may cause damage if they transit beyond the stomach and the ingestion of potential toxins in the food; (3) preliminary reports of vomiting- or regurgitation-like behavior in several species of cephalopod and indirect supporting evidence from the fossil record of regurgitolites; and (4) a conceptual model showing that all the key mechanisms exist in cephalopods (exemplified particularly by *O. vulgaris*) by which ejection of gastric/crop contents could occur.

We fully recognize that the evidence presented is largely circumstantial and is not conclusive, but we reiterate that we have been unable to identify any publications, which refute the ability of cephalopods to either vomit or regurgitate. However, we consider that on balance the limited evidence supports our hypothesis that cephalopods are likely to possess the ability to either vomit or regurgitate; so how can definitive data to confirm or refute our hypothesis be obtained using currently available methodology?

- Vomiting and regurgitation may be rare events. Obtaining proof may be challenging as the ejection event is likely to be brief (a few seconds) and while the animal may adopt a characteristic posture or exhibit prodromal behavior as occurs in vertebrates (see chapter 8 in Stern et al., 2011), it may attempt to hide while doing this to make itself less vulnerable to predation. The arms may also obscure direct observation of the beak to observe ejection. To gather data to better assess its occurrence (or not), we request that the readers of this paper send reports of what they consider to be vomiting- or regurgitation-like behavior in cephalopods in either the wild or captivity. Additionally, Citizen Science Programs (such as the Cephalopod Citizen Science project – https://www.researchgate.net/project/Cephalopod-Citizen-Science) can be used as a resource to search historical social media posts or to request video that may possess evidence of cephalopods vomiting from the SCUBA community.
- Analyze long duration video recordings of *Nautilus*, cuttlefish, and octopus post feeding in captivity to identify vomiting- or regurgitation-like events to provide more robust evidence in one or more species of cephalopod. Collection and analysis of the suspected vomit is essential to understanding the function of vomiting or regurgitation (should either occur) in cephalopods. Detailed analysis of the fecal composition of cephalopods fed on natural diets known to contain indigestible residues and diets containing indigestible artificial markers will enable firmer conclusions to be drawn about how the digestive tract in cephalopods handles indigestible food residues. Studies of *Nautilus* will be particularly important because of its ancestral role for the most recent coleoid cephalopods (e.g., Tanner et al., 2017) and the clear descriptions of relatively large pieces of indigestible material in the stomach from the original description by Owen (1832) to more recent authors (Haven, 1972).
- Undertake *in vitro* studies using isolated digestive tracts to investigate the presence of sphincters between the esophagus/crop/stomach, the existence of retrograde contractile activity in the esophagus, crop, and stomach, and investigate the potential role of the gastric ganglion in switching motility in the upper digestive tract from a digestive pattern to a vomiting or regurgitation pattern. The above studies would use a combination of studies on isolated strips already used in squid and octopus digestive tract (Wood, 1969; Andrews and Tansey, 1983), video recording and quantification of movements in the isolated digestive tract (as exemplified by Figures 2, 3) but using more sophisticated analytical techniques, such as those used in fish and mammalian intestine (e.g., Kendig et al., 2016;  Brijs et al., 2017), and finally recording of intra-luminal pressure, a technique widely used in vertebrates including fish (e.g., Andrews and Young, 1993).
- *In vivo* studies may be necessary to provide definitive proof of the existence of vomiting or regurgitation. Among the mammals, although the ability to vomit is widespread, it is not present in rodents and lagomorphs (Sanger et al., 2011; Horn et al., 2013), so it would be unwise to extrapolate either positive or negative findings from one cephalopod group to another. Studies of multiple species of cephalopod should only be contemplated if studies outlined in i–iii above are not definitive as otherwise it will not be possible to demonstrate to an Ethical Review Committee that there is no alternative to *in vivo* studies. In the European Union, it is a requirement of the legislation (2010/63/EU) regulating animal experimentation (see Fiorito et al., 2015, for details) that all alternative methods, which could answer the scientific question posed have been explored before undertaking *in vivo* studies that meet the threshold for a regulated procedure (see Cooke et al., 2019, for details). By analogy with other experimental procedures in cephalopods, we consider that *in vivo* experiments to demonstrate vomiting or regurgitation would be prospectively classed as “moderate severity” under 2010/63/EU (see Cooke et al., 2019, for details). Studies to demonstrate vomiting or regurgitation face two practical challenges:
  1. *Reliable induction of vomiting or regurgitation*. In the section on “Triggering the Process” above, we identified potential pharmacological stimuli. The study would require administration of a range of substances intravenously (e.g., Agnisola et al., 1996) or by direct administration to the crop/stomach by gavage (e.g., Berk et al., 2009; Sykes et al., 2017) or inclusion in the food. The study would need to be designed to minimize the number of animals required to identify the dose effective in all animals (ED_100_).
  2. *Ensuring the origin of ejected material*. The mouth is obscured by the arm crown making observation of the final ejection of digestive tract contents problematic, and also ensuring that the ejected material originated from the crop/stomach rather than from residues in the buccal cavity or trapped in the arm crown or arm web. Delivering food (e.g., pieces of fish, mussel, or cephalopod) directly into the stomach mixed with an indigestible radio-opaque or fluorescent marker would permit monitoring of the appearance (either by vomiting, regurgitation, or defecation) of food and the indigestible marker in the water in close proximity to the animal. Marking food ingested by the animal with a “dye,” which changes color when exposed to mildly acidic pH (5–6) and proteases would avoid the need for gavage. To permit monitoring the animal would need to be adapted to a relatively small tank during study, and hence, such studies would be difficult to perform in squid.

### What if Cephalopods Lack the Ability to Vomit?

Although we consider it likely that at least some species of cephalopod (e.g., *Nautilus*) can vomit or regurgitate upper digestive tract contents, the possibility remains that they do not have this ability, so here we discuss the implications if this is the case in one or more species.

If cephalopods lack the ability to vomit or regurgitate, then the digestive tract must be able to break down indigestible food residues sufficiently for them to pass through all post-gastric parts of the digestive tract without damaging the mucosa or producing an obstruction of the intestine (particularly where it narrows due to the typhlosole) with the animal finally able to pass the material *via* the anal sphincter. However, vomiting is also one of the mechanisms by which organisms eject contaminated food to reduce the systemic toxic load, so if cephalopods are unable to either vomit or regurgitate then how could they defend themselves against food containing toxins?

An insight into the above question comes from mammals. As far as is known, among the mammals, rodents and lagomorphs are unique in lacking an ability to vomit accounted for by anatomical constraints, differences in brainstem pathways integrating and coordinating the motor outputs, and the motilin system compared to species with an emetic reflex (Sanger et al., 2011; Horn et al., 2013). Rodents do however possess well-developed conditioned aversive responses particularly involving taste (conditioned taste aversion, CTA); following ingestion of a substance presumed to induce the sensation of nausea (or a functionally equivalent sensation), they will avoid ingestion of that substance when presented on a future occasion. It is argued that the taste of the food, and probably also the smell, appearance, and place where it was eaten, is linked to the learned aversion and subsequent avoidance by the sensation of nausea (or other negative hedonic sensation) following ingestion. CTA also occurs in mammalian species with an emetic reflex, including humans (Stern et al., 2011). In fish, learned aversion provides a mechanism by which they avoid poisonous corals (Gerhart, 1984, 1991). Aversive aspects of consumer-prey interactions in marine organisms are reviewed in detail by Paul et al. (2007) and Sotka et al. (2009). Is there evidence for chemoreception and learned aversive responses in cephalopods?

There is evidence, particularly from cuttlefish and octopus, that chemoreceptors are present on the suckers, the lips, and in the olfactory organs (Hanlon and Messenger, 2018, for overview). Distance chemoreception has been implicated in food detection (e.g., Boyle, 1983, 1986; Chase and Wells, 1986; Lee, 1992) and reproductive behavior (e.g., Cummins et al., 2011; Polese et al., 2015). Reviewing behavioral studies of the ability of *O. vulgaris* to discriminate between solutions of sucrose, hydrochloric acid, quinine, and sea water with potassium chloride, Hanlon and Messenger (2018, p. 31) commented “octopuses can probably detect quite small differences in the taste of objects that they handle.” The potential role of either type of chemoreceptor in detection of toxins in the food prior to ingestion (c.f., taste in mammals) remains to be investigated but two relevant studies are discussed below.

Darmaillacq et al. (2004) in adult *S. officinalis* provided preliminary evidence for taste aversion learning in cephalopods by investigating the response of cuttlefish to crabs painted with quinine, perceived by humans as bitter tasting. After eight trials, the cuttlefish learned to avoid the crabs painted with quinine, suggesting that they had developed a learned aversion. The sensation experienced by the cuttlefish is presumed to be one with negative connotations (c.f., pain). However, it must be noted that the animals did not ingest the crab, so the situation differs from the mammalian studies in which animals ingest the contaminated food and link the negative hedonic experience (nausea or equivalent) to the taste, smell, sight of food, or the place where it was ingested. Despite the significant differences in protocol, the Darmaillacq et al. (2004) study does show that cuttlefish are able to learn to avoid a specific food based upon a negative sensory experience. Similar studies in *O. vulgaris* did not provided any evidence of taste aversion (Zarrella and Ponte, personal communication).

*O. vulgaris* will reject pieces of sardine marinated in 3% quinine hydrochloride (c.f., above cuttlefish study) when it comes into contact with the suckers, whereas untreated sardine was accepted (Altman, 1971). The posterior buccal lobe of the inferior frontal region controls the rejection response (Altman, 1971). The chemosensory apparatus in the suckers of cephalopods is likely to act as the first line of defense against the ingestion of noxious material and *O. vulgaris* can be trained to distinguish between hydrochloric acid, sucrose, and quinine solutions applied to the suckers (Wells, 1963). However, the range of substances to which the suckers are sensitive has not been explored using the type of molecular techniques, which have provided insights into taste transduction in mammals. For example, bitter tastants, such as quinine, act *via* the T2R receptor family in mammals (Chandrashekar et al., 2000). Studies are needed to investigate the presence of this G-protein coupled receptor (GPCR) receptor family in cephalopods (e.g., see Ritschard et al., 2019).

Further studies are required to investigate whether cephalopods can learn to avoid foods, particularly those frequently containing toxins or pathogens, which produce “illness” following ingestion. In species with a relatively narrow dietary range becoming averted to one or more of the main types of prey (e.g., crabs, mussels, or fish) may be disadvantageous, so cephalopods may lack this capability and may rely instead on the ability of the digestive gland for metabolic detoxification (Bustamante et al., 2002; Penicaud et al., 2017; Rodrigo and Costa, 2017). It is also possible that toxins in the food may be degraded by salivary enzymes with which the food is mixed during external digestion and ingestion [for review of cephalopod salivary glands see Ponte and Modica (2017)].

For a detailed discussion of strategies adopted by predators in enabling them to deal with foods, which are “chemically defended,” the reader is referred to the review by Glendinning (2007). A detailed review of the role of chemoreception in prey detection and food ingestion in cephalopods is required to contribute to the discussion of whether they have the capacity for learned avoidance of potentially toxic prey, which may obviate part of the need to either vomit or regurgitate. Additionally, a detailed examination of the expression of taste receptor molecules in the suckers would give insights into the spectrum of chemosensitivty.

## Conclusion

Collation of diverse indirect evidence from the literature, a consideration of digestive tract morphology, innervation and physiology, and limited laboratory observations leads us to propose that at least some species of cephalopod are likely to be capable of either vomiting or regurgitation. Reviewing the evidence has identified a number of gaps in knowledge of the anatomy (e.g., the presence of sphincters) and physiology (e.g., the fate of indigestible food residues, pH of digestive secretions, and digestive gland detoxification mechanisms) as well as the properties and functions of epithelial chemoreceptors. The capacity of cephalopods to either vomit or regurgitate, or neither, now requires more formal investigation. Such studies should form part of a wider consideration of other adaptations, which may enable cephalopods to identify (e.g., vision and chemosensitivity) and avoid (learned aversion) potentially toxic foods, neutralize ingested toxins (e.g., salivary and digestive glands) and deal with indigestible material (e.g., gizzard-like stomach in octopus).

## Data Availability Statement

The datasets generated for this study will not be made publicly available. There are no data that could be considered a “data set” that would be of use to others. Also, the previously unpublished data included is preliminary. The videos are available *via* a link but they are not a “dataset.”

## Ethics Statement

Ethical review and approval was not required for the animal study because the novel behavioral data reported on “live cephalopods” are observations made during routine husbandry and care and are neither experiments or regulated procedures (as defined in 2010/63/EU). The *in vitro* observations are from tissue removed *post mortem* and are not regulated by 2010/63/EU. The manuscript includes a note to this effect where the previously unpublished findings, which support the hypothesis, are described.

## Author Contributions

All authors contributed to the article and approved the submitted version.

### Conflict of Interest

The authors declare that the research was conducted in the absence of any commercial or financial relationships that could be construed as a potential conflict of interest.

## Footnotes

^1^https://youtu.be/0tFz2ppx_DY; https://youtu.be/c02EZcjWNSE

^2^https://youtu.be/Pymql7ncWjI
